# Assessment and Prognostic Value of Immediate Changes in Post-Ablation Intratumor Density Heterogeneity of Pulmonary Tumors *via* Radiomics-Based Computed Tomography Features

**DOI:** 10.3389/fonc.2021.615174

**Published:** 2021-11-03

**Authors:** Bo Liu, Chunhai Li, Xiaorong Sun, Wei Zhou, Jing Sun, Hong Liu, Shuying Li, Haipeng Jia, Ligang Xing, Xinzhe Dong

**Affiliations:** ^1^ Department of Radiology, Qilu Hospital, Cheeloo College of Medicine, Shandong University, Jinan, China; ^2^ Department of Radiology, Shandong Cancer Hospital and Institute, Jinan, China; ^3^ Department of Radiation Oncology, Qilu Hospital, Cheeloo College of Medicine, Shandong University, Jinan, China; ^4^ Key Laboratory of Biobased Polymer Materials, Shandong Provincial Education Department, College of Polymer Science and Engineering, Qingdao University of Science and Technology, Qingdao, China; ^5^ Department of Radiation Oncology, Shandong Cancer Hospital and Institute, Shandong University, Jinan, China

**Keywords:** lung neoplasm, computed tomography (CT), radiomics, response assessment, microwave ablations (MWA)

## Abstract

**Objectives:**

To retrospectively observe the instantaneous changes in intratumor density heterogeneity after microwave ablation (MWA) of lung tumors and to determine their prognostic value in predicting treatment response and local tumor progression (LTP).

**Methods:**

Pre- and post-MWA computed tomography (CT) images of 50 patients (37-males; 13-females; mean-age 65.9 ± 9.7y, 39 primary and 11 metastasis) were analyzed to evaluate changes in intratumor density. Global, regional, and local scale radiomics features were extracted to assess intratumor density heterogeneity. In four to six weeks, chest enhanced CT was used as the baseline evaluation of treatment response. The correlations between the parametric variation immediately after ablation and the visual score of ablation response (Rvisu) were analyzed by nonparametric Spearman correlation analysis. The 1-year LTP discrimination power was assessed using the area under the receiver operating characteristic (ROC) curves. A Cox proportional hazards regression model was used to identify the independent prognostic features.

**Results:**

Although no significant volume changes were observed after ablation, the radiomics parameters changed in different directions and degrees. The mean intensity value from baseline CT image was 30.3 ± 23.2, and the post-MWA CT image was -60.9 ± 89.8. The ratio of values change was then calculated by a unified formulation. The largest increase (522.3%) was observed for cluster prominence, while the mean CT value showed the largest decline (321.4%). The pulmonary tumors had a mean diameter of 3.4 ± 0.8 cm. Complete ablation was documented in 36 patients. Significant correlations were observed between Rvisu and quantitative features. The highest correlations were observed for changes in local features after MWA, with r ranging from 0.594 to 0.782. LTP developed in 22 patients. The Cox regression model revealed Δcontrast% and response score as independent predictors (Δcontrast%: odds ratio [OR]=5.61, p=0.001; Rvisu: OR=1.73, p=0019). ROC curve analysis showed that Δcontrast% was a better predictor of 1-year LTP. with higher sensitivity (83.5% vs. 71.2%) and specificity (87.1% vs. 76.8%) than those for Rvisu.

**Conclusions:**

The changes in intratumor density heterogeneity after MWA could be characterized by analysis of radiomics features. Real-time density changes could predict treatment response and LTP in patients with pulmonary tumors earlier, especially for tumors with larger diameters.

## Introduction

Primary lung cancer is the most common cancer and the leading cause of cancer-related mortality in China and worldwide (1). The abundant blood supply and special growth microenvironment in lung tissue favor metastatic development. Owing to this permissive environment, the lungs are one of the most common sites of metastases, with 30% of patients with malignant solid tumors developing pulmonary metastases (2).

In addition to systemic therapy, the principal local therapies for lung cancer and lung metastases include surgical resection, external-beam radiotherapy, and thermal ablation procedures such as radiofrequency ablation (RFA) and microwave ablation (MWA) (3). Compared to RFA, MWA is reportedly a more effective treatment for lung tumors (4–6).

MWA has reported rates of complete ablation of 80–90%, with the best results obtained for tumors <3 cm in diameter. For tumors >3 cm, multiple points or treatments can be delivered, and/or other treatments can be used in combination (7). However, the clinical efficacy of MWA for larger pulmonary tumors has not yet been determined (8). Chest enhanced computed tomography (CT) examinations are often performed 4–6 weeks after the procedure to evaluate the local efficacy of the treatment. However, the earlier evaluation of the local efficacies of MWA could allow more timely interventions, such as second MWA and radiation therapy, which might help to reduce tumor load and prolong overall survival (9).

The immediate change in intratumor density after MWA may also indicate the treatment response or long-term outcomes. Recently, radiomics studies, which extract a large number of quantitative features from medical images, have been proposed for various clinical applications (10–13). For lung cancer, our previous studies found that changes in metabolic tumor heterogeneity during concurrent chemoradiotherapy (CCRT), characterized by global and local scale textural features, may provide independent information to predict treatment response and survival (14).

However, to our knowledge, there have been no reports of changes in the tissue density heterogeneity features from CT imaging of lung tumors in patients receiving MWA. Therefore, the present study performed dynamic radiomics analysis of CT imaging to retrospectively observe the early changes in intratumor density heterogeneity after MWA of the lung and to determine their prognostic value in predicting treatment and local tumor progression (LTP).

## Materials and Methods

### Patients

This study was approved by the Institutional Review Board of the Ethics Committee of Qilu Hospital of Shandong University. Written informed consent was obtained from all participating patients before they underwent CT-guided percutaneous MWA of their lung tumors. Patients were recruited based on the following eligibility criteria (1): patients (aged ≥18 years) who were referred to our radiology department for MWA of lung tumors (2); malignant lung tumors confirmed by histological or cytological diagnosis (3); re-examination by chest enhanced CT 4–6 weeks after the procedure, which was used as the baseline for evaluation (4); tumor volume ≥2.5 cm^3^; and (5) adequate normal organ function. Patients were excluded if they had undergone radiotherapy or other local or regional treatments before or after MWA. A cohort of 50 patients (39 primary and 11 metastatic) who had received MWA for a lung tumor between November 2017 and September 2019 were retrospectively identified for study inclusion.

### Imaging Protocol and MWA

Immediately before the intervention, all patients underwent an unenhanced plain chest CT examination (SOMATOM Force, Siemens Healthcare, Germany: 5 mm collimation, 110 reference kVp, and 50 reference mAs using automated tube current modulation). The procedures were performed by a radiologist with over 20 years of experience in thoracic interventions using a set protocol (15). The puncture site and the best entry route for the microwave antenna were determined according to the baseline CT images. The power settings and ablation times were adjusted according to the manufacturer’s protocols (4–7 min for 3.7 cm active tip at 60 W, 4–7 min for a 3.2 cm active tip at 55 W or 50 W, and 4–6 min for a 2.7 cm active tip at 45 W, respectively) while considering the tumor size and location. Lidocaine was administered at the puncture site to induce local anesthesia of the pleura. With CT monitoring, MWA was performed using a microwave applicator (ECO-100A1, Microwave Electronic Institute, Nanjing, China) at 2.45 GHz (for medical use). The applicator included a 16-gauge straight microwave antenna (10, 15, or 20 cm in length). A peristaltic pump perfused the outer shaft of the antenna with normal saline at 60 mL/min to prevent thermal injury along the proximal antenna shaft. Considering tumor shape and size, one or two needle ablations with a constant antenna position were usually adequate to achieve complete ablation of small lesions (<3.0 cm) or medium-sized tumors (3.0-5.0 cm). After completion of the MWA session, the ablation antenna was removed. A repeat CT (same parameters) scan was performed immediately after the ablation to assess the occurrence of ablation-related complications and whether the tumor had been fully covered by the ablation.

### Immediate Assessment of Radiomics Changes

We selected the delineation method based on its availability and clinical usefulness. All lesions were manually contoured by a single experienced radiologist who was blinded to the patients’ history. The borders of region of interest (ROI) were set by manually with lung window (window width and level:1500, -500). The ROIs were checked and validated by an independent senior radiologist. The 3D volume was then segmented and used for comparative analysis. The entire dataset of the treatment planning and post-MWA non-contrast-enhanced CT images, all acquired with a 5-mm slice thickness, were analyzed to extract textural features from the ROIs. Larger tumor volume could provide adequate information. Influence of body posture during CT scans was very small.

Following manual delineation, the structure of ROI and all CT images were transferred to Matlab software (Matrix Laboratory, MathWorks Inc., Natick, USA) using the Digital Imaging and Communications in Medicine (DICOM) protocol. The main image processing methods such as ROI segmentation, denoising, and radiomics feature extraction were performed using in-house MATLAB code. Global, regional, and local scale textural features were extracted from grey-volume histogram analysis and normalized gray-level co-occurrence matrix (NGLCM), run-length matrix (RLM), and neighborhood intensity difference matrix (NIDM), respectively, to assess intratumor density heterogeneity. All Texture features were derived from the 3D imaging. These parameters have been widely used in CT and robustly depict global intratumor heterogeneity (14, 16, 17). The parameters in planning and post-ablation CT scans were labeled as P1 and P2, respectively. The immediate assessment of radiomics change in percentage (ΔP) was calculated as [(*P*2–*P*1/*P*1*100%. The post-treatment value subtracted by baseline, and then divided by baseline. Negative values represent a decrease of in ROIs. Positive values indicate an increase of CT parameters.

### Complications and Short-Term Evaluation of the Local Efficacy

All complications are classified according to the time of occurrence and imaging findings. Chest enhanced CT was re-examined 4–6 weeks after the procedure and used as the baseline for evaluation. Complications were graded according to the criteria of the International Working Group on Image-guided Tumor Ablation under the Society of Interventional Radiology (SIR).

The two outcomes of interest in this study were the short-term evaluation of local efficacy and LTP. Modified Response Evaluation Criteria in Solid Tumors (mRECIST) were used to evaluate the local efficacy. Three radiologists with different levels of experience independently evaluated the first post-ablation enhanced CT images without knowledge of the patients’ clinical history or subsequent follow-up. Two lesion characteristics—size and enhanced zone of the CT image—were evaluated and numerically graded (**Supplementary Data 1**). Based on the mRECIST criteria, complete ablation (without an enhanced zone) was scored as 5 for volume shrinkage, 4 for unchanged or slightly enlarged volume; 3 for incomplete ablation (remaining enhanced zone of <50% of the baseline CT), 2 for remaining enhanced zone ≥50% of the baseline CT, and 2 for local progression (newly developed enhanced zone). The mean value of the three observers was used to assess the local efficacy.

Follow-up ended with the most recent clinic visit, most recent imaging study before September 2020, or the patient’s death. LTP was defined as the development of a new enhancing tumor during follow-up after documentation of technical success.

### Statistical Analysis

Statistical analyses were performed using IBM SPSS for Windows, version 23.0 (IBM Corp., Armonk, NY). Data are presented as means ± standard deviation (SD). The differences between P1 and P2 were defined using Wilcoxon signed-rank or paired t-tests after confirming the normal distribution of the parameters by Shapiro–Wilks tests. The correlations between heterogeneity parameters and response scores were analyzed using nonparametric Spearman correlations. LTP was defined as the main endpoint to evaluate the prognostic value of the parameters. Patients who underwent a second MWA or radiology treatment before LTP development were censored from the LTP evaluation on the treatment day. Survival curves were generated using the Kaplan–Meier method. The differences in survival rates among the groups were compared using log-rank tests. Multivariate analysis was performed to identify independent prognostic factors using a Cox proportional hazards regression model. The 1-year LTP discrimination power was assessed based on area under the receiver operating characteristic (ROC) curve analysis. All statistical tests were conducted at a two-sided significance level of P<0.05.

## Results

### Patient Characteristics

Between November 2017 and September 2019, 50 consecutive patients (mean age, 65.9 y; age range, 52–82 years) with 50 lung lesions, including metastases and primary lung cancers, underwent MWA. These lesions had received no pre-MWA or post-MWA local therapies, including surgery or radiotherapy. No severe lung infections or other intraprocedural complications occurred in any patient during the follow-up. Mild pneumothorax, pleural effusion, and pulmonary hemorrhage occurred in five patients and resolved within a short time. The average ablation time was 3.7 ± 0.6 min (power, 51.4 ± 3.1 w). The patient demographics and clinical characteristics are shown in **Table 1**.

**Table 1:** Patient demographic and clinical characteristics.

Patient characteristic		Number of cases (%)
Age	≥65	32 (64%)
	<65	18 (36%)
Gender	Male	37 (74%)
	Female	13 (26%)
Tumor location	upper and middle lobes	27 (54%)
	right lobe	23 (46%)
Tumor depth	central	9 (18%)
	peripheral	41 (82%)
Maximum diameter (cm)	2-3cm	11 (22%)
	3-5cm	31 (62%)
	>5cm	8 (16%)
Primary pulmonary lesion	adenocarcinoma	21 (42%)
	squamous cell carcinoma	15 (30%)
	small cell carcinoma	1 (2%)
	others	2 (4%)
Metastatic lesion from	colorectal cancer	3 (6%)
	breast cancer	2 (4%)
	kidney cancer	1 (2%)
	sarcoma	2 (4%)
	esophageal	2 (4%)
	carcinoma, unknown primary	1 (2%)
Complications	pneumothorax	3 (6%)
	pleural effusion	1 (2%)
	pulmonary hemorrhage	2 (4%)

### Changes in Tissue Density After MWA

No significant changes in two-axis measurements (long- and short-axis diameters) and ablation zone volume were observed immediately after high- or low-dose lung tissue ablation. The pulmonary tumors had a mean diameter of 3.4 ± 0.8 cm (range 2.4–5.3 cm) at baseline with no significant change after MWA. The planned pathway from the skin to lesion had a mean length of 8.1 ± 2.6 cm (range of 3.3–11.0 cm). The baseline tumor volume was 5.5 ± 7.9 cm^3^. No significant differences were observed in any shape parameters, including sphericity, elongation, flatness, etc.

Changes in intratumor density change were evaluated post-MWA by CT. To quantify the changes in tissue density, a series of 3D radiomics features at different scales were recorded. Paired t-test analysis revealed significant changes in some local, regional, and global parameters, while the other parameters were not normally distributed. The mean intensity decreased 321.4 ± 187.3% (P=0.000). The radiomics parameters changed in different directions and degrees, as shown in **Table 2**. Overall, the largest increase (522.3 ± 223.7%) was observed for cluster prominence (glcm) while the mean CT value (first order) showed the largest decrease.

**Table 2:** Immediate change of tumor density after MWA characterized by radiomics features.

Parameters		Mean value (before MWA)	Mean value (Immediate after MWA)	Ratio of change	P Value
Global	Mean	30.342	-60.893	-3.214 ± 1.873	0.000
	Medium	24.182	-26.653	-2.296 ± 2.035	0.000
	10 Percentile	14.702	51.235	0.802 ± 2.926	0.000
	Robust Mean Absolute Deviation	42.670	57.098	0.380 ± 0.878	0.002
	Kurtosis	9.155	14.770	0.340 ± 1.659	0.012
	Variance	13397.636	18877.005	0.279 ± 1.328	0.035
Regional	Zone Percentage	0.191	0.312	0.494 ± 0.765	0.000
	Small Area Emphasis	0.506	0.755	0.335 ± 0.751	0.027
	Large Area Emphasis	6526.487	4211.368	-0.398 ± 0.419	0.001
	Short Run Low Gray Level Emphasis	0.015	0.020	0.317 ± 0.256	0.032
	Long Run Low Gray Level Emphasis	0.275	0.307	0.372 ± 0.214	0.045
Local	Cluster Prominence	2703.426	16633.482	5.223 ± 2.237	0.000
	Maximum Probability	0.202	0.125	-0.448 ± 0.429	0.001
	Cluster Tendency	41.367	102.460	0.577 ± 1.806	0.000
	Joint Energy	0.079	0.047	-0.432 ± 0.579	0.015
	Contrast	16.206	31.968	0.415 ± 1.178	0.000
	Joint Entropy	5.588	6.559	0.259 ± 0.336	0.001
	Correlation	0.395	0.524	0.223 ± 0.697	0.004
	Sum Squares	14.259	29.878	0.517 ± 1.597	0.000

### MWA Efficacy

**Figure 1:**
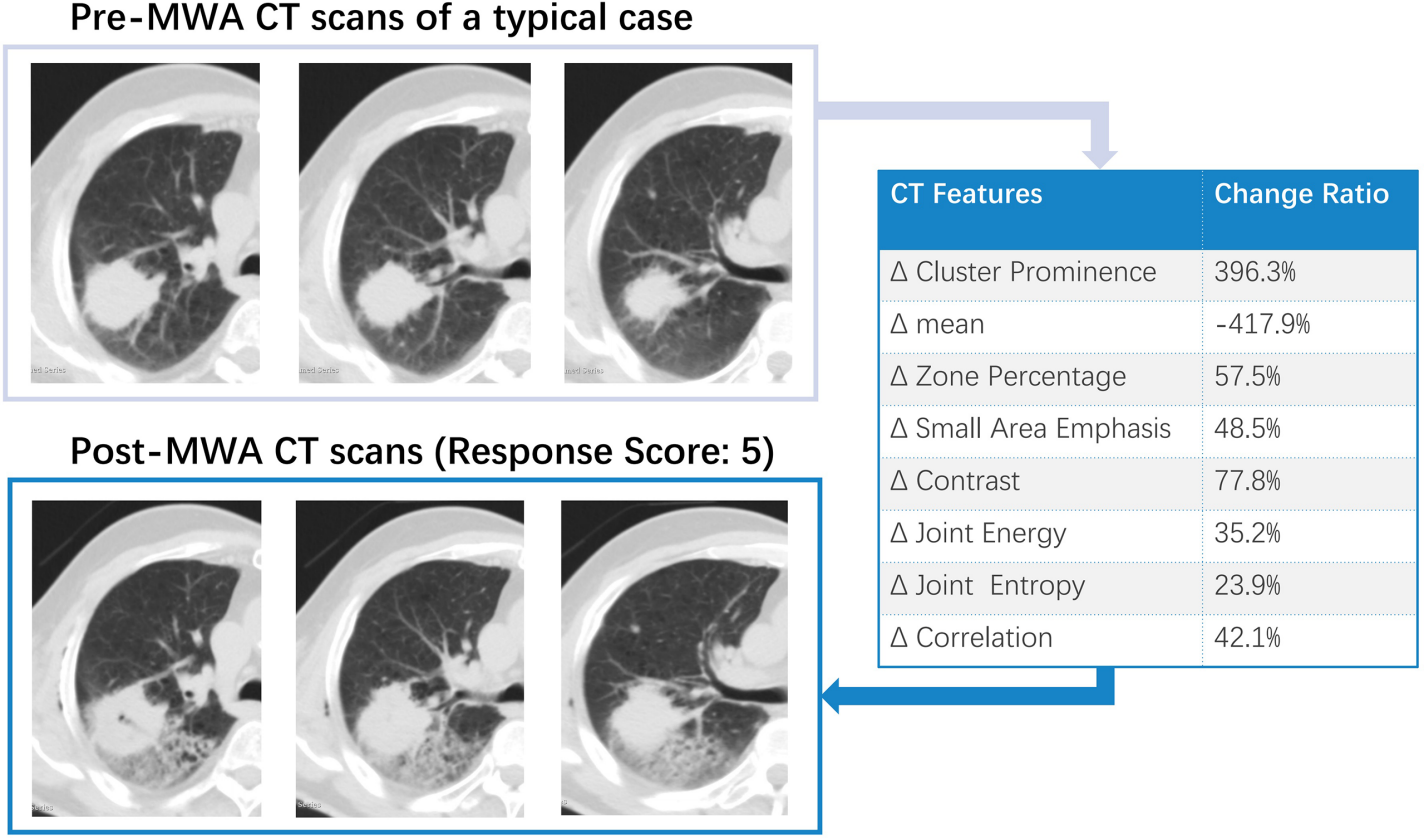
A typical example of intra-tumoral tissue density features’ change in CT image for patient with complete ablation. Pre-and Post-MWA image showed high repeatability. The subsequent effect of registration error was largely avoided in the data analysis.

**Figure 2:**
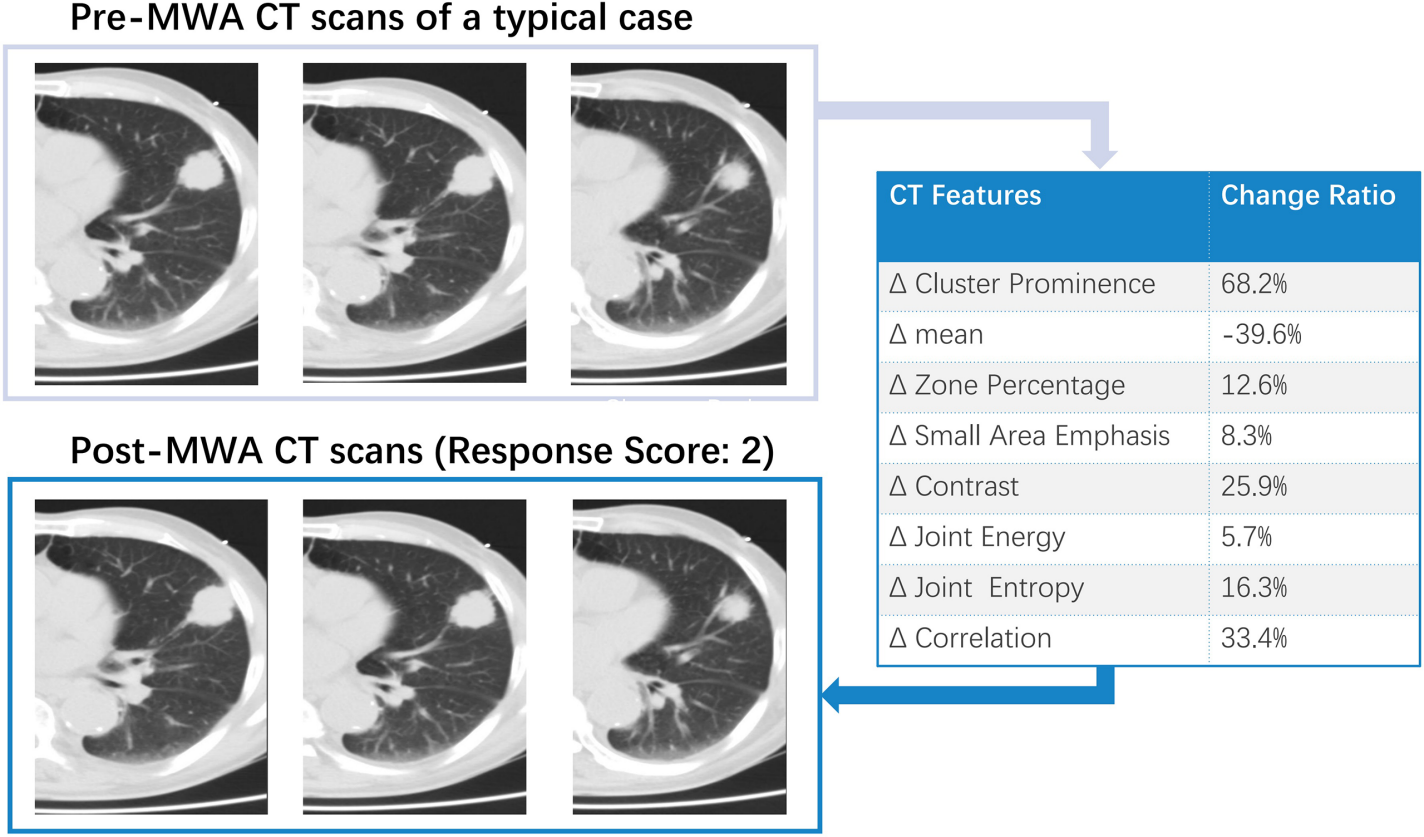
A typical example of intra-tumoral tissue density features’ change in CT image for patient with incomplete ablation.

All patients underwent follow-up contrast-enhanced CT at 1 month (median interval, 28 days; range, 22–39 days). Two patients (2/50, 4%) showed a viable larger tumor on the edge of the ablative margin; the others (48/50, 96%) did not show any evidence of a new residual viable tumor. Complete ablation was documented in 36 patients (72%), including 20 patients (40%) with visual scores of 5 and 16 patients (32%) with scores of 4. Twelve patients were classified as having incomplete ablation (Rvisu=3: 9 patients; Rvisu=2: 3 patients). **Figures 1**, **2** show typical examples of heterogeneity change in intratumor density in the CT images of patients with complete and incomplete ablation.

### Correlations Between Visual Scoring and Quantitative Changes in Intratumor Density

**Figure 3:**
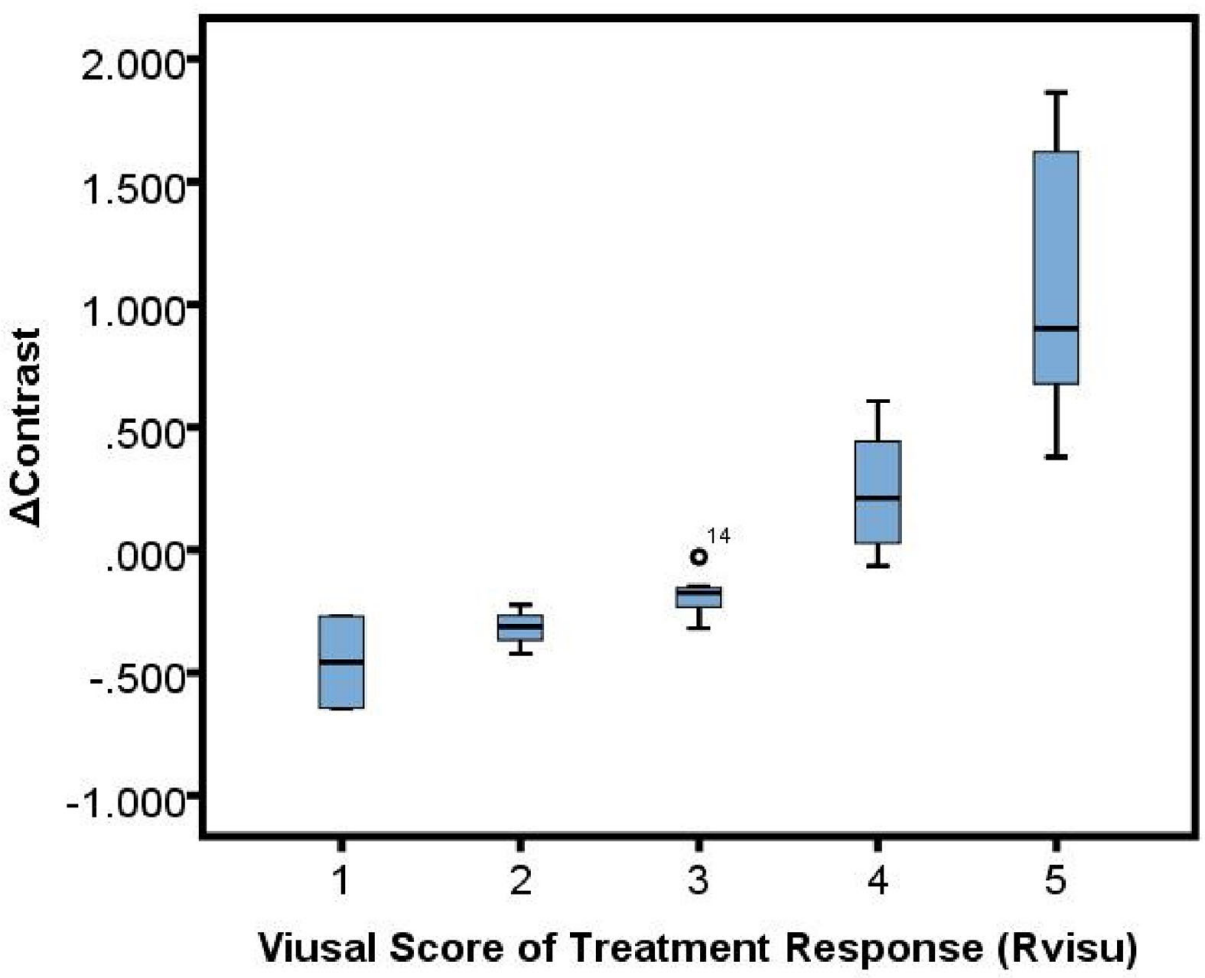
Box plot showed a linear correlation between ΔContrast% and Rvisu.

Significant correlations were observed between Rvisu and quantitative features (**Table 3**). The highest correlations were observed for changes in local features after MWA, with r values ranging from 0.594 to 0.782, except for cluster prominence, maximum probability, and correlation (r=0.426, P=0.032; r=0.362, P=0.017; and r=0.507, P=0.062, respectively). The box plots showed a linear correlation between ΔContrast and Rvisu (**Figure 3**). Except for the zone percentage (r=0.625, P=0.003), other regional parameters were not correlated with Rvisu (P>0.05). Whereas mean and medium CT value changes showed significant changes after MWA, only mean value and kurtosis were correlated with tumor response (r=0.504, P=0.038 and r=0.472, P=0.017, respectively). In contrast, baseline tumor volume and diameter showed variable levels of correlation with tumor response. These correlations showed that, although ablation efficacy was correlated with volume, such changes in intratumor density could provide complementary information.

**Table 3:** The relationships between tumor response (Rvisu) and quantitative features.

Parameters		r	P value
Baseline	Diameter	0.624	0.001
	Volume	0.712	0.000
Global	Δmean	0.504	0.038
	ΔKurtosis	0.472	0.017
Regional	ΔZone Percentage	0.625	0.003
Local	ΔCluster Tendency	0.594	0.000
	ΔJoint Energy	0.439	0.023
	ΔContrast	0.782	0.000
	ΔJoint Entropy	0.526	0.012
	ΔSum Squares	0.491	0.037

### LTP After MWA

**Figure 4:**
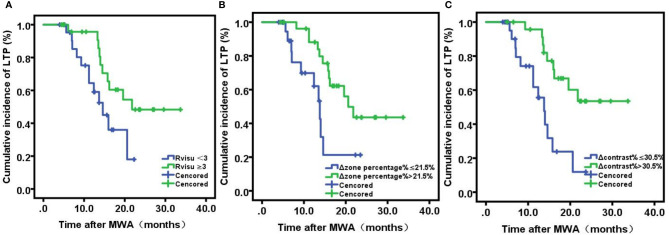
Kaplan-Meier analyses of local tumor progression. **(A)** Viusal Score; **(B)** ΔZone percentage%; **(C)** Δ: contrast%.

The median follow-up interval was 19 months (range, 12–28). During the follow-up period, 22 patients (44%) developed LTP. Patients with sufficient ablative margins (R_visu_≥3) showed a significantly lower cumulative incidence of LTP compared to patients with insufficient ablative margins (38.5% [10/26] vs. 50% [12/24], P=0.036) (**Figure 4A**). Kaplan–Meier analysis showed that among baseline tumor features, only tumor volume was a statistically significant prognostic factor for LTP. LTP was shorter in patients with lower Δzone percentage% (median LTP 9.4 months vs. 16.5 months, P=0.15) (**Figure 4B**). In addition, Δcontrast% >30.5% was significantly associated with improved LTP (median LTP: 17.2 months vs. 10.3 months, P=0.001) (**Figure 4C**). Because of the high degree of collinearity among the various image features, each was entered separately in the multivariate Cox regression model, which revealed Δcontrast% and response score as independent predictors (Δcontrast%: odds ratio [OR]=5.61, 95% confidence interval [CI]:1.458–12.85, P=0.001; Rvisu: OR=1.73, 95% CI: 0.72–10.68, P=0.019), while other clinical features were not significant predictors of survival (**Table 4**). In addition, ROC curve analysis showed that Δcontrast% was a better predictor of 1-year LTP, with higher sensitivity (83.5% vs. 71.2%) and specificity (87.1% vs. 76.8%) than baseline parameters or Rvisu.

**Table 4:** Results of Uni- and multi-variate analyses for predictors of LTP in 50 patients.

Parameters	Univariate Analysis		Multivariate Analysis	
	Odds Ratio (95% CI)	P	Odds Ratio (95% CI)	P
Sex (male)	0.94 (0.83–1.08)	0.076		
Age (≥67)	0.72 (0.28–4.36)	0.421		
Smoker	1.42 (0.53–3.73)	0.164		
Diameter (≥3.6cm)	0.91 (0.68–1.42)	0.053		
Volume (≥4.1cm^3^)	0.56 (0.27–1.52)	0.066		
Rvisu (≥3)	1.68 (0.54–9.72)	0.036	1.63 (0.72 –10.68)	0·019
Δcontrast%>30.5%	6.59 (1.83–27.2)	0.001	5.61 (1.458-12.85)	0.001
Δzone percentage%>21.5%	2.56 (0.25–3.4)	0.015	1.95 (0.68–1.42)	0.072

## Discussion

MWA has attracted increasing attention as a minimally invasive therapy. Thus, there is a growing need to establish not only the optimal follow-up imaging protocol but also an imaging reference for assessing treatment response. To date, no standard follow-up imaging protocol has been established or uniformly accepted (18). Re-examination by chest enhanced CT is most commonly performed 4–6 weeks after the procedure and is used as the baseline for evaluation. The results of the present study showed that intratumor real-time density changes predicted treatment response and LTP earlier, especially for tumors with larger diameters.

Significant changes in the ablation zone may occur because of its role in dehydration, protein denaturation, and contraction of collagen. Radiomics approaches have been applied to quantify changes in intratumor density heterogeneity during ablation, which may ultimately correlate with clinical outcomes in a variety of tumors (16, 19, 20). Elena et al. (21) designed a three-dimensional quantitative ablation assessment technique to accurately access the minimum margin size, which showed higher discrimination power compared to the 2D method (AUC 0.893 vs. 0.790 (p = 0.01). Volumetric assessment of the ablation zone in the liver is feasible and can improve the accuracy of 2-year LTP prediction following thermal ablation of hepatic tumors. Our observations in the present study are consistent with these previous findings. In lung cancer, our previous research suggested that early changes in metabolic tumor heterogeneity (defined by global and local scale textural features) during chemoradiotherapy served as a response predictor with higher sensitivity (73.2–92.1%) and specificity (80.0–83.6%) compared to baseline parameters (14). Based on these results, we explored the early changes in tissue density heterogeneity characterized by radiomics features after MWA, to non-invasively capture intratumor heterogeneity.

While challenging, obtaining additional biometric information can be rewarding (22). A growing number of studies have reported the usefulness of quantitative imaging features to characterize malignant lung tumors based on other imaging modalities such as magnetic resonance imaging (MRI) and positron emission tomography (PET) (23). In a study analyzing the role of MRI for the early evaluation of lung MWA, Andrei et al. (24). retrospectively investigated the images from 49 patients who underwent unenhanced chest CT and contrast-enhanced MRI (ceMRI) (including T2 and ceT1) 24 h after MWA. The MRI sensitivity and specificity for pneumothorax were 60.8% and 87.0%, respectively, and MRI showed a similar ability to predict LTP and detect important complications as CT. In their study, changes in radiomics features showed better performance than conventional contrast CT performed 1 month after MWA. Researchers have also evaluated the utility of ^18^F-fluorodeoxyglucose positron emission tomography/computed tomography (18F-FDG PET/CT) for response assessment. Singnurkar et al. (25) reported that 18F-FDG PET/CT parameters on both pre- and post-ablation scans predicted local recurrence in patients treated with RFA for lung metastases and primary lung cancers. However, post-ablation PET/CT scans were performed within 1–4 months after therapy, an economic burden that may be too high for most patients.

Early detection of the risk of recurrence is critical, especially for larger tumors. The real-time evaluation of tissue reactions is necessary to predict treatment response. Moreover, the early identification of residual tumor or recurrence could allow timely retreatment or alternative therapy. The mean tumor diameter in this study was 3.4 cm. Patients with tumors sized ≤3 cm may respond best to ablation. The local control rate decreased when the tumor size was >3 cm. Based on these findings, we included more larger tumors. In addition, for precision radiomics analysis, higher voxel numbers were needed, such that small tumors could not provide sufficient information for further analysis.

Recently, Yoon et al. (26) evaluated the clinical impact of using registration software for ablative margin assessment on pre-RFA MRI and post-RFA CT compared to the conventional side-by-side MR-CT visual comparison. The main limitation was that the pre-RFA MRI was performed on two different scanners with different parameters, including slice thicknesses, which may have affected the image registration quality. As shown in our investigation, the two-set CT series were obtained using the same position and scan parameters. **Figures 1**, **2** show the high repeatability and reliability of this method. Thus, the effects of registration errors were largely avoided in the data analysis. To date, no standard follow-up imaging protocol or quantitative indicators have yet been established. Although a gold standard radiomic pattern for tumor characterization remains challenging, our results hold great promise.

Significant changes in the ablation zone may occur because of its role in dehydration, protein denaturation, and collagen contraction. However, studies have reported conflicting observations of tissue characteristics following ablation procedures, including tissue shrinkage (e.g., 15–50%) or an increase in the ablation zone in the first 24 hours (27). This study did not consider inflammatory response, which may be a potential limitation. However, the focus was the immediate response of tumor tissue, which could reflect the biological behavior of the tumor in the absolute sense. Kodama et al. (28). investigated the development and evolution of MWA lesions in the normal lung using a swine model at various time points (immediately post-treatment and at 2, 7, 14, and 28 days). They found that the treatment zone following MWA in the lung varied in the sub-acute setting, peaking in area at 7 days post-treatment. Furthermore, CT measurements closely matched the gross pathologic ablation size. These results supported our findings that temporal CT imaging density reflected the immediate response of tumor cells. Based on this observation, radiologists should pay more attention to post-MWA CT findings, which could help us to discover more hidden information and identify patients requiring early interventions.

Owing to its limitation, the results of this study must be interpreted with care. One major limitation was the relatively small number of patients. Considering the limited local treatment effect of ablation, we enrolled both primary and metastatic tumors. Future studies will include larger sample sizes and different pathologies. We found no significant difference between primary and metastasis in post-treatment change of this study. Moreover, additional studies with more patients are also needed to validate the texture analysis for this application. Another limitation of our study was that we only analyzed larger tumors. However, given the limited spatial resolution and sickness of planning CT imaging, the assessment of tissue structure heterogeneity in small structures may provide little useful information. Moreover, the CT protocols and microwave applicator may not have been identical between subjects and between the two hospitals where the CT images were acquired, which may have affected the texture analysis. However, we use the change rate of radiomics features to counteract this influence. In addition, we included a heterogeneous study population, including both primary and metastatic lesions. Further studies are necessary to evaluate the predictive performance of post-ablation CT.

## Conclusion

Immediate assessment of the changes in tissue density heterogeneity between pre- and post-ablation CT imaging may provide independent information to predict treatment response and survival in patients with pulmonary tumors.

## Data Availability Statement

The original contributions presented in the study are included in the article/**Supplementary Material**. Further inquiries can be directed to the corresponding author.

## Ethics Statement

The studies involving human participants were reviewed and approved by Ethics Committee of Qilu Hospital of Shandong University. The patients/participants provided their written informed consent to participate in this study.

## Author Contributions

BL and CL: Methodology, Writing-Reviewing and Editing; XS: Writing- Original draft preparation; WZ: Software; JS: Validation; HL: Investigation; SL: Software; HJ: Data curation; LX: Visualization; XD: Conceptualization and Supervision.

## Funding

This work was supported by grants from Shandong Provincal Nature Science Foundation (Project ZR2019LZL007 and ZR2018BH029), as well as Shandong Provincal Key Research and Development Program (Project 2018GSF118087).

## Conflict of Interest

The authors declare that the research was conducted in the absence of any commercial or financial relationships that could be construed as a potential conflict of interest.

## Publisher’s Note

All claims expressed in this article are solely those of the authors and do not necessarily represent those of their affiliated organizations, or those of the publisher, the editors and the reviewers. Any product that may be evaluated in this article, or claim that may be made by its manufacturer, is not guaranteed or endorsed by the publisher.

## References

[1] Xue X Asuquo I Hong L Gao J Dong Z Pang L. Catalog of Lung Cancer Gene Mutations Among Chinese Patients. Front Oncol (2020) 10:1251. doi: 10.3389/fonc.2020.01251 32850378 PMC7417348

[2] Németh T Szabó Z Pécsy B Barta ZV Lázár G Torday L. Changes in the Surgical Treatment of Pulmonary Metastases During the Last 12 Years. Orv Hetil (2020) 161:1215–20. doi: 10.1556/650.2020.31770 32628621

[3] Londero F Grossi W Morelli A Parise O Masullo G Tetta C. Surgery Versus Stereotactic Radiotherapy for Treatment of Pulmonary Metastases. A Systematic Review of Literature. Future Sci OA (2020) 6:FSO471. doi: 10.2144/fsoa-2019-0120 32518686 PMC7273364

[4] Nian-Long L Bo Y Tian-Ming C Guo-Dong F Na Y Yu-Huang W. The Application of Magnetic Resonance Imaging-Guided Microwave Ablation for Lung Cancer. J Cancer Res Ther (2020) 16:1014–9. doi: 10.4103/jcrt.JCRT_354_20 33004742

[5] Ni Y Xu H Ye X. Image-Guided Percutaneous Microwave Ablation of Early-Stage Non-Small Cell Lung Cancer. Asia Pac J Clin Oncol (2020) 16:320–5. doi: 10.1111/ajco.13419 32969192

[6] Brace CL Hinshaw JL Laeseke PF Sampson LA Lee FT Jr. Pulmonary Thermal Ablation: Comparison of Radiofrequency and Microwave Devices by Using Gross Pathologic and CT Findings in a Swine Model. Radiology (2009) 251:705–11. doi: 10.1148/radiol.2513081564 PMC2687532 19336667

[7] Liu BD Ye X Fan WJ Li XG Feng WJ Lu Q. Expert Consensus on Image-Guided Radiofrequency Ablation of Pulmonary Tumors: 2018 Edition. Thorac Cancer (2018) 9:1194–208. doi: 10.1111/1759-7714.12817 PMC6119618 30039918

[8] Prud'homme C Teriitehau C Adam J Kyaw Tun J Roux C Hakime A. Lung Microwave Ablation - An *In Vivo* Swine Tumor Model Experiment to Evaluate Ablation Zones. Int J Hyperthermia (2020) 37:879–86. doi: 10.1080/02656736.2020.1787530 32689829

[9] Wang Y Li G Li W He X Xu L. Radiofrequency Ablation of Advanced Lung Tumors: Imaging Features, Local Control, and Follow-Up Protocol. Int J Clin Exp Med (2015) 8:18137–43. PMC4694311 26770411

[10] Chetan MR Gleeson FV. Radiomics in Predicting Treatment Response in Non-Small-Cell Lung Cancer: Current Status, Challenges and Future Perspectives. Eur Radiol (2020) 31:1049–58. doi: 10.1007/s00330-020-07141-9 PMC7813733 32809167

[11] Li J Li X Chen X Ma S. Research Advances and Obstacles of CT-Based Radiomics in Diagnosis and Treatment of Lung Cancer. Zhongguo Fei Ai Za Zhi (2020) 23:904–8. doi: 10.3779/j.issn.1009-3419.2020.101.36 PMC7583873 32798440

[12] Avanzo M Stancanello J Pirrone G Sartor G. Radiomics and Deep Learning in Lung Cancer. Strahlenther Onko (2020) 196:879–87. doi: 10.1007/s00066-020-01625-9 32367456

[13] Wang C Dong X Sun X Zhang R Xing L. Association of Radiomic Features With Epidermal Growth Factor Receptor Mutation Status in Non-Small Cell Lung Cancer and Survival Treated With Tyrosine Kinase Inhibitors. Nucl Med Commun (2019) 40:1091–8. doi: 10.1097/MNM.0000000000001076 31469811

[14] Dong X Sun X Sun L Maxim PG Xing L Huang Y. Early Change in Metabolic Tumor Heterogeneity During Chemoradiotherapy and Its Prognostic Value for Patients With Locally Advanced Non-Small Cell Lung Cancer. PLoS One (2016) 11:e0157836. doi: 10.1371/journal.pone.0157836 27322376 PMC4913903

[15] Jia H Tian J Liu B Meng H Pan F Li C. Efficacy and Safety of Artificial Pneumothorax With Position Adjustment for CT-Guided Percutaneous Transthoracic Microwave Ablation of Small Subpleural Lung Tumors. Thorac Cancer (2019) 10:1710–6. doi: 10.1111/1759-7714.13137 PMC6669918 31290286

[16] Dong X Xing L Wu P Fu Z Wan H Li D. Three-Dimensional Positron Emission Tomography Image Texture Analysis of Esophageal Squamous Cell Carcinoma: Relationship Between Tumor 18F-Fluorodeoxyglucose Uptake Heterogeneity, Maximum Standardized Uptake Value, and Tumor Stage. Nucl Med Commun (2013) 34:40–6. doi: 10.1097/MNM.0b013e32835ae50c 23111378

[17] Ferreira Junior JR Koenigkam-Santos M Cipriano F Fabro AT Azevedo-Marques PM. Radiomics-Based Features for Pattern Recognition of Lung Cancer Histopathology and Metastases. Comput Methods Programs Biomed (2018) 159:23–30. doi: 10.1016/j.cmpb.2018.02.015 29650315

[18] Hendriks P Noortman WA Baetens TR van Erkel AR van Rijswijk C van der Meer RW. Quantitative Volumetric Assessment of Ablative Margins in Hepatocellular Carcinoma: Predicting Local Tumor Progression Using Nonrigid Registration Software. J Oncol (2019) 2019:4049287. doi: 10.1155/2019/4049287 31641353 PMC6770329

[19] Forghani R Savadjiev P Chatterjee A Muthukrishnan N Reinhold C Forghani B. Radiomics and Artificial Intelligence for Biomarker and Prediction Model Development in Oncology. Comput Struct Biotechnol J (2019) 17:995–1008. doi: 10.1016/j.csbj.2019.07.001 31388413 PMC6667772

[20] Dong X Sun X Zhao X Zhu W Sun L Huang Y. The Impact of Intratumoral Metabolic Heterogeneity on Postoperative Recurrence and Survival in Resectable Esophageal Squamous Cell Carcinoma. Oncotarget (2017) 8:14969–77. doi: 10.18632/oncotarget.14743 PMC5362458 28122340

[21] Fouladi DF Zarghampour M Pandey P Pandey A Varzaneh FN Ghasabeh MA. Baseline 3d-ADC Outperforms 2D-ADC in Predicting Response to Treatment in Patients With Colorectal Liver Metastases. Eur Radiol (2020) 30:291–300. doi: 10.1007/s00330-019-06289-3 31209620

[22] Du D Feng H Lv W Ashrafinia S Yuan Q Wang Q. Machine Learning Methods for Optimal Radiomics-Based Differentiation Between Recurrence and Inflammation: Application to Nasopharyngeal Carcinoma Post-Therapy PET/CT Images. Mol Imaging Biol (2020) 22:730–8. doi: 10.1007/s11307-019-01411-9 31338709

[23] Chen J Lin ZY Wu ZB Chen ZW Chen YP. Magnetic Resonance Imaging Evaluation After Radiofrequency Ablation for Malignant Lung Tumors. J Cancer Res Ther (2017) 13:669–75. doi: 10.4103/jcrt.JCRT_448_17 28901312

[24] Roman A Kaltenbach B Gruber-Rouh T Naguib NN Vogl TJ Nour-Eldin NA. The Role of MRI in the Early Evaluation of Lung Microwave Ablation. Int J Hyperthermia (2018) 34:883–90. doi: 10.1080/02656736.2017.1377354 28877612

[25] Singnurkar A Solomon SB Gönen M Larson SM Schöder H. 18f-FDG PET/CT for the Prediction and Detection of Local Recurrence After Radiofrequency Ablation of Malignant Lung Lesions. J Nucl Med (2010) 51:1833–40. doi: 10.2967/jnumed.110.076778 21078787

[26] Yoon JH Lee JM Klotz E Woo H Yu MH Joo I. Prediction of Local Tumor Progression After Radiofrequency Ablation (RFA) of Hepatocellular Carcinoma by Assessment of Ablative Margin Using Pre-RFA MRI and Post-RFA CT Registration. Korean J Radiol (2018) 19:1053–65. doi: 10.3348/kjr.2018.19.6.1053 PMC6201982 30386137

[27] Rossmann C Garrett-Mayer E Rattay F Haemmerich D. Dynamics of Tissue Shrinkage During Ablative Temperature Exposures. Physiol Meas (2014) 35:55–67. doi: 10.1088/0967-3334/35/1/55 24345880 PMC3924587

[28] Kodama H Ueshima E Howk K Lee SW Erinjeri JP Solomon SB. Temporal Evaluation of the Microwave Ablation Zone and Comparison of CT and Gross Sizes During the First Month Post-Ablation in Swine Lung. Diagn Interv Imaging (2019) 100:279–85. doi: 10.1016/j.diii.2018.10 30581098

