# Supplementary material for: Assessment and Prognostic Value of Immediate Changes in Post-Ablation Intratumor Density Heterogeneity of Pulmonary Tumors via Radiomics-Based Computed Tomography Features
Source: Front Oncol. 2021 Nov 3;11:615174. doi: 10.3389/fonc.2021.615174 (PMC8595917; doi:10.3389/fonc.2021.615174)
Supplement: Supplementary file 1 [file Table_1.docx]

Supplementary Table Scoring System based on modified RECIST

| Effectiveness | Score | CT (change of size & density change) |
| --- | --- | --- |
| Complete ablation | 5 | Without an enhanced zone, volume shrinks |
|  | 4 | Without an enhanced zone, volume unchanged or enlarged slightly |
| Incomplete ablation | 3 | Enhanced zone ＜50% of baseline CT |
|  | 2 | Enhanced zone ≥50% of baseline CT |
| Local progression | 1 | Newly developed enhanced zone |
